# Changes in Bulk and Rhizosphere Soil Microbial Diversity and Composition Along an Age Gradient of Chinese Fir (*Cunninghamia lanceolate*) Plantations in Subtropical China

**DOI:** 10.3389/fmicb.2021.777862

**Published:** 2022-02-23

**Authors:** Yuzhe Wang, Pengyu Jiao, Wen Guo, Dajun Du, Yalin Hu, Xiang Tan, Xian Liu

**Affiliations:** ^1^College of Forestry, Fujian Agriculture and Forestry University, Fuzhou, China; ^2^Key Laboratory of Aquatic Botany and Watershed Ecology, Wuhan Botanical Garden, Chinese Academy of Sciences, Wuhan, China

**Keywords:** rhizosphere, microbial community, stand age, 16S and ITS *r*RNA sequencing, Chinese fir plantation

## Abstract

Soil microorganisms play key roles in biogeochemical cycling in forest ecosystems. However, whether the responses of microbial community with stand development differed in rhizosphere and bulk soils remains unknown. We collected rhizosphere and bulk soil in Chinese fir plantations with different stand ages (7a, 15a, 24a, and 34a) in subtropical China, and determined bacterial and fungal community variation *via* high-throughput sequencing. The results showed that soil bacterial, but not fungal, community diversity significantly differed among stand ages and between rhizosphere and bulk soils (*p* < 0.05). The differences in Shannon–Wiener and Simpson’s indices between rhizosphere and bulk soil varied with stand age, with significant higher soil bacterial diversity in rhizosphere than bulk soils in 7a and 34a plantations (*p* < 0.05), but there were no significant difference in soil bacterial diversity between rhizosphere and bulk soils in 15a and 24a plantations (*p* > 0.05). Soil microbial community composition varied significantly with stand age but not between the rhizosphere and bulk soil. The dominant bacterial phyla at all ages were *Acidobacteria* and *Proteobacteria*, while the dominant fungal phyla were *Ascomycota* and *Basidiomycota* in both rhizosphere and bulk soil. They showed inconsistent distribution patterns along stand age gradient (7–34a) in the rhizosphere and bulk soil, suggesting distinct ecological strategy (r-strategist vs. k-strategist) of different microbial taxa, as well as changes in the microenvironment (i.e., nutrient stoichiometry and root exudates). Moreover, bacterial and fungal community composition in rhizosphere and bulk soil were governed by distinct driving factors. TP and NH_4_^+^–N are the two most important factors regulating bacterial and fungal community structure in rhizosphere soil, while pH and NO_3_^–^–N, DON, and TN were driving factors for bacterial and fungal community structure in bulk soil, respectively. Collectively, our results demonstrated that the changes in microbial diversity and composition were more obvious along stand age gradients than between sampling locations (rhizosphere vs. bulk soil) in Chinese fir plantations.

## Introduction

Chinese fir [*Cunninghamia lanceolata* (Lamb.) Hook] is an endemic, evergreen coniferous timber species and widely cultivated in southern China because of its high yield, rapid growth rate, and excellent wood quality. Despite the long planting history (>1,000 years) and large cultivation area (>12 × 10^6^ ha) in China (Chen et al., 2013), current intensive management practice (e.g., successive crops of Chinese fir at the same site, whole-tree harvesting, etc.) and shortened rotation time (20–25 years) have been reported to cause a serious decline in forest productivity (Yang et al., 2004; Wu et al., 2017). A previous study reported that the growth of Chinese fir was limited by nitrogen (N) in subtropical China (Tong et al., 2020), and there was a shift from N limitation to phosphorus (P) limitation with the stand development of Chinese fir plantations (Wu et al., 2020). It seems that the reason for productivity decline in Chinese fir plantation is related to the converted nutrient cycling process, while the underlying microbial mechanism still remains unclear.

Plant–microbe interactions are central to soil fertility, biogeochemical cycling, and associated ecosystem functions. On one hand, microbial communities influence plant growth and soil nutrient cycling in forest ecosystems (Philippot et al., 2013; Liu et al., 2018); on the other hand, plants have strong selective effects on soil microbial communities *via* specific litter chemistry (Urbanova et al., 2015; Ferreira et al., 2017). The rhizosphere is a hotspot for microbial–plant interactions, with much of the activity involving exchange of energy and nutrients (Hinsinger et al., 2009). In contrast, bulk soil has relatively oligotrophic conditions, with low rates of nutrient transformation and microbial activity (Ai et al., 2012). A recent meta-analysis showed that rhizosphere effect of woody plants could accelerate the biogeochemical cycling in rhizosphere soil and increased soil C and nutrient pool (Gan et al., 2021). Moreover, the distributional patterns and drivers of microbial communities may differ between rhizosphere and bulk soil (Fan et al., 2018; Liu et al., 2018). Previous studies reported that changes in litter composition and soil nutrient stoichiometry were significant predictors for bulk microbial communities (Sun et al., 2016; Li et al., 2017), while rhizosphere microorganisms were strongly affected by tree species, root exudates, soil and root properties, and plant growth stages (Delgado-Baquerizo et al., 2017).

Several studies have reported the changes in soil properties of Chinese fir plantation induced by different stand age (Wu et al., 2011; Zhang et al., 2016; Selvaraj et al., 2017); however, knowledge of the influence of stand age on soil bulk and rhizosphere microbial communities is still limited. During the development of vegetation, the changes in the composition and diversity of microbial community are likely to be paralleled by shifts in soil physiochemical properties, but whether it is a direct relationship or related to other factors remains uncertain (Liu et al., 2009; Wang et al., 2010; Chen et al., 2020). Hence, understanding the coupling relationships among plants, soil parameters, and microbial characteristics along an age gradient of Chinese fir plantation provides insight into the response mechanisms of soil microbes during vegetation developing process.

In general, the changes in microbial communities are regulated by many biotic and abiotic factors. Specifically, biotic factors, such as plant functional traits, species richness, and community diversity, have been demonstrated as critical predictors for microbial communities at the regional level (Lange et al., 2015; Prober et al., 2015). Moreover, abiotic factors, such as soil pH (Wang et al., 2015) and nutrient levels (Ren et al., 2018), were reported to be key regulators shaping the structures of microbial communities. Zhong et al. (2019) reported that soil physiochemical properties, especially soil organic carbon, total nitrogen, NO_3_^–^–N and bulk density, play an important role in determining soil microbial diversity and composition along an age gradient of *Pinus tabulaeformis* stands in the Loess Hilly Region of China. Xu et al. (2019) found that soil N-fixing microbial communities generally varied from *Actinobacteria*-dominated cropland to *Proteobacteria*-dominated forest soil with stand ages in a *Robinia pseudoacacia* chronosequence, and the variation was tightly linked to the level of N fractions.

In this study, we investigated the distributional patterns and dominant factors influencing the bacterial and fungal communities in the rhizosphere and bulk soil along an age gradient of from 7 to 34 years of Chinese fir stands. We hypothesized that the (1) changes in different stand ages would make an impact in soil microbial communities, including changes in abundance and community diversity, and (2) these changes would be related to distinct factors in the rhizosphere and bulk soil, and fungal and bacterial communities, respectively. Therefore, the main objectives of our study were to (1) examine the changes in microbial communities in rhizosphere and bulk soil along an age gradient in Chinese fir plantation, (2) explore whether changes in soil properties and plant characteristics have different impacts on fungal and bacterial community structure, and (3) distinguish the predominant driving factors of the shifts in soil microbial community composition and diversity along an age gradient in Chinese fir plantation.

## Materials and Methods

### Experimental Site and Soil Sampling

The study site was located in the Xiqin forest farm of Fujian Agriculture and Forestry University, Nanping City, Fujian Province, China (26°33′N, 118°6′E). This area is characterized by subtropical monsoon climate, with mean annual temperature of 19.4°C, mean annual precipitation of 1,817 mm, and a frost-free period of 302 days. The soil is Ferric Acrisol under the World Reference Base soil classification system (IUSS Working Group WRB, 2015). The total plantation area of the forest farm is 1,190 ha, and the plantation area accounts for 46%.

The space-for-time method was used to investigate the changes in soil microbial community diversity and composition with stand development of Chinese fir plantations. Four Chinese fir monoculture plantations with different stand ages (7, 15, 24, and 34 years) but with similar soil types, elevation, slope degree, and aspect were selected. In August 2019, four replicated plots of 20 × 20 m were randomly set up in each age group, and the replicate plots were more than 30 m apart from each other. There were totally 16 plots in this study. At each plot, three trees with similar diameter at breast height (DBH) that represented the mean level of the stand were selected, and both rhizosphere and bulk soils were collected. Rhizosphere soils were defined as those tightly adhered to the root surface and collected after shaking the roots (Phillips and Fahey, 2006), and the root-free soil was collected as bulk soil. A total of 96 samples were collected (2 types of samples × 4 stand ages × 4 replicate plots × 3 trees). The fresh soil samples were sealed in plastic bags and transported to the laboratory as soon as possible. After removing stones and other debris, soils were sieved through a 2-mm mesh and divided into three subsamples. One subsample was stored at 4°C for the analysis of moisture, dissolved organic C (DOC), and available N. Another subsample was air dried for the analysis of pH, total C (TC), total N (TN), total P (TP), and available P (AP), and the rest was frozen at −80°C for soil DNA extraction and microbial community analysis.

### Soil Physiochemical Analysis

Soil pH was measured with a pH meter using a soil-to-water ratio of 1:2.5 (w/v). Moisture content was determined gravimetrically after drying the soil at 105°C for 24 h. TC and TN were measured using Elemental Analyzer (Vario MICRO cube, Elementar, Germany). After extracting with 2 M KCl, soil NH_4_^+^–N and NO_3_^–^–N content in the suspension was measured by Smartchem Discrete Auto Analyzer (AMS, Italy). DOC and total soluble N (TSN) were measured by a TOC-VCPH/CPN analyzer (Shimadzu Scientific Instruments, Japan) after extracting by 2 M KCl using a soil-to-water ratio of 1:5. Dissolved organic N (DON) was calculated by the differences between TSN and total inorganic N (NH_4_^+^–N + NO_3_^–^–N). TP was digested with H_2_SO_4_–HClO_4_, and AP was extracted by 0.5 M NaHCO_3_ solution, colored by a molybdenum–antimony solution, and measured using the spectrophotometric method (Olsen et al., 1954).

### DNA Extraction, Amplification, and MiSeq Sequencing

DNA was extracted from 0.3 g of soil using the MoBio PowerSoil™ DNA Isolation Kit (MO BIO Laboratories, Carlsbad, CA, United States) following the instructions of the manufacturer. The quantity and quality of the extracted DNA were determined using a DeNovix DS-11 spectrophotometer (DeNovix, Wilmington, DE, United States).

The primers of 515F (5′-GTGCCAGCMGCCGCGGTAA-3′) and 806R (5′-GGACTACHVGGG TWTCTAAT-3′) were used to amplify the V3–V4 region of bacterial 16S *r*RNA gene (Zhang et al., 2015), and the fungal internal transcribed spacers (ITSs) were amplified using the eukaryote-specific primers ITS5-1737F (5′-GGAAGTAAAAGTCGTAACAAGG-3′) and ITS2-2043R (5′-GCTGCGTTCTTCATCGATGC-3′) (White et al., 1990). PCR reactions were carried out with 15 μl of Phusion^®^ High-Fidelity PCR Master Mix (New England Biolabs, Ipswich, United Kingdom), 0.2 μM forward primer, 0.2 μM reverse primer, and about 10 ng of template DNA. The PCR products were purified with Qiagen Gel Extraction Kit (Qiagen, Hilden, Germany). The high-throughput sequencing was performed on an Illumina NovaSeq platform provided by Novogene Bioinformatics Technology Co., Ltd., Beijing, China.

### Processing of Sequencing Data

The Uparse software (Uparse v7.0.1001^1^) was used to assign sequences with more than 97% similarity to the same operational taxonomic units (OTUs) (Edgar, 2013). Silva reference database^2^ was used to determine the taxonomic assignment of each representative sequence. The sequencing data have been deposited in the National Center for Biotechnology Information (NCBI) Sequence Read Archive (SRA) database under accession number PRJNA694295. Alpha diversity indices including Shannon–Wiener and Simpson indices were calculated with QIIME (Version 1.7.0) (Caporaso et al., 2010).

### Statistical Analysis

The normality of all data was checked and met before statistical analysis. Two-way analyses of variance (ANOVAs) were used to analyze the effects of plantation age, soil location (bulk soil and rhizosphere) and their interaction on the soil properties, and microbial attributes (number of OTUs, Shannon–Wiener and Simpson’s diversity indices). One-way ANOVA followed by least significant difference (LSD) test was performed to compare the effect of plantation age in rhizosphere or bulk soil when there were significant interactions between sampling location and plantation age. The structure of the bacterial and fungal communities among different plantation ages in the rhizosphere and bulk soils were visualized by non-metric multidimensional (NMDS) based on Bray–Curtis distance using R software with vegan package. We then used analysis of similarities (ANOSIM) to determine differences in soil bacterial and fungal community composition using vegan package. The relative importance of the environmental variables (pH, TC, TN, TP, C:N, C:P, N:P, DOC, DON, DOC:DON, NH_4_^+^–N, NO_3_^–^–N, and AP) in explaining the microbial community compositions was identified by canonical correspondence analysis (CCA) based on OTU relative abundance data. Pearson correlation analysis was performed to investigate the relationships between soil microbial attributes (OTUs, diversity index) and soil physiochemical properties and nutrient content. Effects of soil properties (soil pH, TC, TN, TP, C:N, C:P, N:P, DOC, DON, DOC:DON, NH_4_^+^–N, NO_3_^–^–N, and AP) on rhizosphere and bulk soil bacterial and fungal community composition were determined by a structural equation model (SEM) using the lavaan package. We constructed an *a priori* model based on our knowledge of the relative contributions and interactions between edaphic factors and microbial community composition. We used the χ^2^ and its associated *p*-value to judge the model fitness. Path standardized coefficient was used to estimate the effect of all the explanatory variables on soil bacterial and fungal community composition. All of the above statistical analyses were conducted with R software (version 3.6.2).

## Results

### Soil Properties Along the Age Gradients

Analyses of variance indicated that most soil parameters were significantly affected by the stand age rather than soil location (rhizosphere vs. bulk soil) and their interactions (stand age × soil location) (Table 1). Plantation age significantly altered all measured soil parameters except for TC, TN, DON, and AP (Table 1, *p* < 0.001), while significant differences were merely observed on soil pH, DOC, DON, and NH_4_^+^–N between rhizosphere and bulk soil (Table 1, *p* < 0.01). As shown in Table 2, all soils were acidic (pH 4.15–4.53), with pH values significantly lower in rhizosphere soil (4.15–4.49) than in bulk soil (4.22–4.53) and higher in young stand age soil (7a) than older stand age soils (15a, 24a, and 34a) (Table 2, *p* < 0.001). Soil NH_4_^+^–N, NO_3_^–^–N, and DON showed uneven trends along with age gradients, being lowest at 15a, 7a for NH_4_^+^–N and NO_3_^–^–N, and 34a for DON (Table 2). The age-related changes in soil DOC differed in rhizosphere and bulk soils, with the value in rhizosphere soil peaking in the 15a plantation, while the highest value for bulk soil observed in the 24a plantation, and soil DOC content in the 34a plantation was the lowest in both rhizosphere and bulk soils. Moreover, soil NO_3_^–^–N followed a decreasing trend from stand age 15a to 34a, which decreased by an average of 30.6, 51.2, and 24.8, 52.1%, respectively, for rhizosphere and bulk soil compared with the 15-year old stand soil. Soil TP was significantly lower at age 34 compared with other stand ages (Table 2, *p* < 0.001), but no significant difference was observed on soil AP among different stand ages (Table 2, *p* > 0.05).

**TABLE 1:** Results of two-way analysis of variance (ANOVA) showing the effects of stand age, location (rhizosphere and bulk), and their interaction on the soil properties, and bacterial and fungal diversity indices.

Factors	pH	Moisture	TC	TN	TP	DOC	DON	NH_4_^+^–N	NO_3_^–^–N	AP
Stand age	***	***	0.28	0.74	***	***	0.27	***	***	0.06
Location	***	0.98	0.19	0.40	0.33	***	**	**	0.75	0.32
Stand age × location	0.46	0.64	0.75	0.82	0.65	***	0.22	0.15	0.96	0.63

		**Bacterial**					**Fungal**		
		
**Number of OTUs**		**Shannon–Wiener diversity**		**Simpson’s diversity**		**Number of OTUs**		**Shannon–Wiener diversity**		**Simpson’s diversity**

0.57		0.19		***		0.46		0.96		0.80
0.07		*		**		0.41		0.13		0.14
0.14		*		*		0.27		0.05		0.05

Numbers in the table indicate “not significant (p > 0.05)”. TC, total carbon content; TN, total nitrogen content; TP, total phosphorus content; DOC, dissolved organic carbon content; DON, dissolved organic nitrogen content; AP, available P content. ***p < 0.001; **p < 0.01; *p < 0.05.

**TABLE 2:** Soil physical and chemical characteristics in the rhizosphere and bulk soil along the age gradient of Chinese fir plantation in subtropical China.

Location	Stand age (years)	pH	Moisture	TC (g kg^–1^)	TN (g kg^–1^)	TP (g kg^–1^)	NH_4_^+^–N (mg kg^–1^)	NO_3_^–^–N (mg kg^–1^)	DOC (mg kg^–1^)	DON (mg kg^–1^)	AP (mg kg^–1^)
Rhizosphere	7	4.44 ± 0.05a	29.75 ± 0.55b	20.15 ± 0.27a	1.32 ± 0.003a	0.30 ± 0.01a	4.74 ± 0.31a	1.46 ± 0.29c	201.58 ± 13.44a	27.15 ± 2.33a	2.28 ± 0.45a
	15	4.29 ± 0.06ab	33.98 ± 1.06a	19.44 ± 0.92a	1.45 ± 0.07a	0.29 ± 0.01a	3.00 ± 0.19b	9.65 ± 1.15a	267.92 ± 24.05a	24.63 ± 4.59a	2.06 ± 0.06a
	24	4.19 ± 0.02c	36.01 ± 1.49a	21.62 ± 1.29a	1.40 ± 0.09a	0.31 ± 0.01a	4.71 ± 0.26a	6.70 ± 1.05ab	112.76 ± 13.87b	30.79 ± 1.46a	1.98 ± 0.19a
	34	4.20 ± 0.05bc	30.12 ± 0.74b	18.62 ± 1.49a	1.32 ± 0.14a	0.23 ± 0.02b	5.48 ± 0.23a	4.71 ± 0.43bc	70.78 ± 14.08b	24.94 ± 2.92a	1.74 ± 0.11a
Bulk	7	4.51 ± 0.02a	30.10 ± 0.68b	19.85 ± 0.54a	1.32 ± 0.04a	0.31 ± 0.02a	6.12 ± 0.47a	1.73 ± 0.39c	70.18 ± 12.74ab	32.26 ± 2.35a	2.69 ± 0.29a
	15	4.46 ± 0.07ab	34.87 ± 0.86a	18.10 ± 0.93a	1.36 ± 0.06a	0.31 ± 0.02a	3.29 ± 0.13b	9.60 ± 0.56a	85.34 ± 7.66ab	42.03 ± 2.69a	2.57 ± 0.45a
	24	4.28 ± 0.06c	34.55 ± 1.34a	19.23 ± 1.45a	1.28 ± 0.06a	0.32 ± 0.005a	5.70 ± 0.11a	7.22 ± 0.78ab	93.15 ± 10.29a	34.93 ± 5.80a	1.84 ± 0.11a
	34	4.41 ± 0.04bc	30.39 ± 0.29b	18.38 ± 1.48a	1.34 ± 0.11a	0.22 ± 0.01b	5.65 ± 0.41a	4.60 ± 0.16bc	54.12 ± 3.42b	28.54 ± 5.09a	1.79 ± 0.29a

*Values are the means ± SE (n = 4). Different letters indicate significant differences among different stand ages in rhizosphere or bulk soil (p < 0.05).*

### Diversity and Composition of Microbial Communities Along the Age Gradients

A total of 1,704,871 and 2,025,211 qualified 16S and ITS sequences were obtained from 32 rhizosphere and bulk soil samples, respectively (Supplementary Table 1). The number of bacterial sequences varied from 34,281 to 69,063 per sample, whereas the number of fungal sequences varied from 52,206 to 69,854 per sample (Supplementary Table 1). These sequences were clustered into OTUs (97% similarity), and the number of bacterial and fungal OTU ranged from 1,905 to 3,066, and 970 to 1962, respectively, depending on different soil samples (Supplementary Table 1). There was no significant effects of stand age and soil location on the number of bacterial and fungal OTUs (Table 3, *p* > 0.05).

**TABLE 3:** Pearson correlation coefficients between bacterial, fungal alpha diversity, and environmental variables.

Soil location	pH	Moisture	TC	TN	TP	C:N	C:P	N:P	DOC	DON	NH_4_^+^–N	NO_3_^–^–N	AP
**Rhizosphere**													
Bacterial—OTUs	0.42	–0.41	–0.44	−−0.63**	–0.21	0.43	–0.14	–0.32	–0.06	–0.13	0.35	−−0.64**	0.02
Bacterial—Shannon–Wiener index	0.29	−0.53*	–0.42	−−0.53*	–0.48	0.29	0.18	0.01	–0.27	–0.01	0.53*	−−0.64**	–0.01
Bacterial—Simpson index	0.13	−−0.55*	–0.31	–0.35	−−0.62**	0.16	0.45	0.3	–0.45	0.07	0.67**	−−0.62*	0.03
Fungal—OTUs	–0.02	–0.03	–0.17	–0.13	0.12	–0.05	–0.27	–0.17	–0.35	–0.19	0.12	0.06	–0.34
Fungal—Shannon–Wiener index	–0.07	–0.14	–0.42	–0.49	–0.24	0.18	–0.05	–0.12	−−0.67**	–0.47	0.6*	–0.29	–0.2
Fungal—Simpson index	0.00	0.00	–0.23	–0.41	–0.09	0.32	–0.06	–0.21	−−0.63**	–0.38	0.57*	–0.35	0.07
**Bulk**													
Bacterial—OTUs	−−0.58*	0.47	–0.45	−−0.5*	0.13	–0.06	–0.42	–0.38	0.59*	0.5	–0.13	0.3	0.13
Bacterial—Shannon–Wiener index	−−0.76***	0.43	–0.37	–0.41	–0.08	–0.06	–0.16	–0.13	0.58*	0.44	–0.04	0.34	–0.12
Bacterial—Simpson index	−−0.7**	0.03	–0.21	–0.28	–0.38	0.05	0.3	0.26	0.21	0.09	0.36	–0.04	–0.29
Fungal—OTUs	0.47	0.00	–0.10	0.00	0.48	–0.13	−−0.52*	–0.42	–0.1	0.08	–0.27	0.15	0.13
Fungal—Shannon–Wiener index	0.46	–0.01	–0.04	–0.04	0.52*	0.01	−−0.53*	–0.49	0.03	0.01	–0.12	0.02	0.15
Fungal—Simpson index	0.46	–0.03	–0.03	–0.02	0.32	–0.01	–0.35	–0.33	0.07	–0.03	–0.07	–0.08	0.21

Bold * and ** indicate significant at P < 0.05 and 0.01.

**FIGURE 1:**
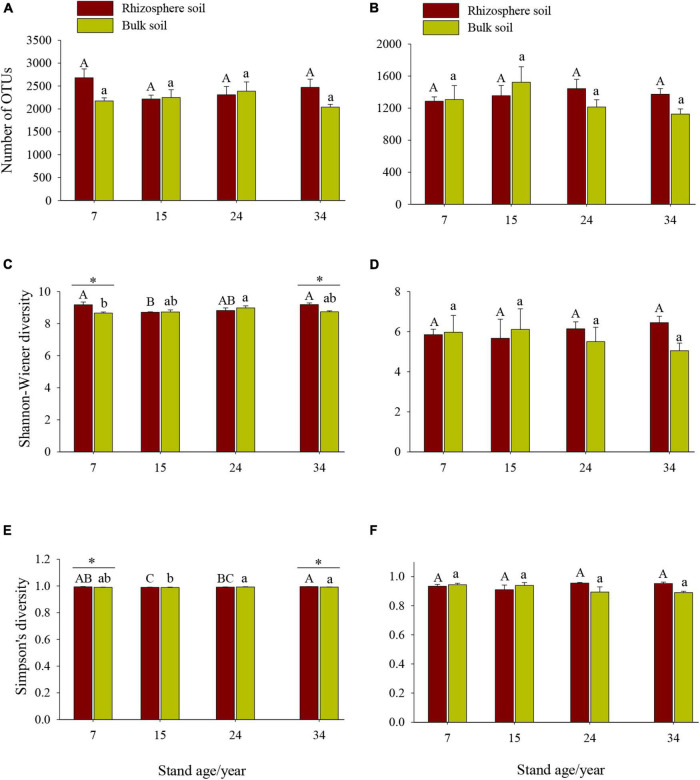
Differences in bacterial **(A,C,E)** and fungal **(B,D,F)** alpha diversity among different stand ages in Xinkou Forest Farm, Southeastern China. Data are means ± SE (*n* = 4). Different uppercase and lowercase letters represent significant differences (*p* < 0.05) among different stand ages within rhizosphere and bulk soil, respectively; *significant differences between rhizosphere and bulk soils based on a one-way analysis of variance (ANOVA) followed by a least significant difference (LSD) test (*p* < 0.05).

Stand age, soil location, and their interactions significantly influence the Shannon–Wiener and Simpson’s indices of soil bacterial, but not fungal, community (Figure 1 and Table 3). For the bacterial community, the Shannon–Wiener and Simpson’s indices indicated significant differences along the chronosequence and differed in rhizosphere and bulk soils, with significant higher bacterial Simpson’s indices in rhizosphere soil of the 34a plantation than those in the 15a plantation (Figure 1, *p* < 0.001). The significant differences in Shannon–Wiener and Simpson’s indices between rhizosphere and bulk soil varied with stand age, with significant higher soil bacterial diversity in rhizosphere than bulk soils in the 7a and 34a plantations (Figures 1C,E, *p* < 0.05), while no significant differences in soil bacterial diversity between rhizosphere and bulk soils in the 15a and 24a plantations were observed (Figures 1C,E, *p* > 0.05).

**FIGURE 2:**
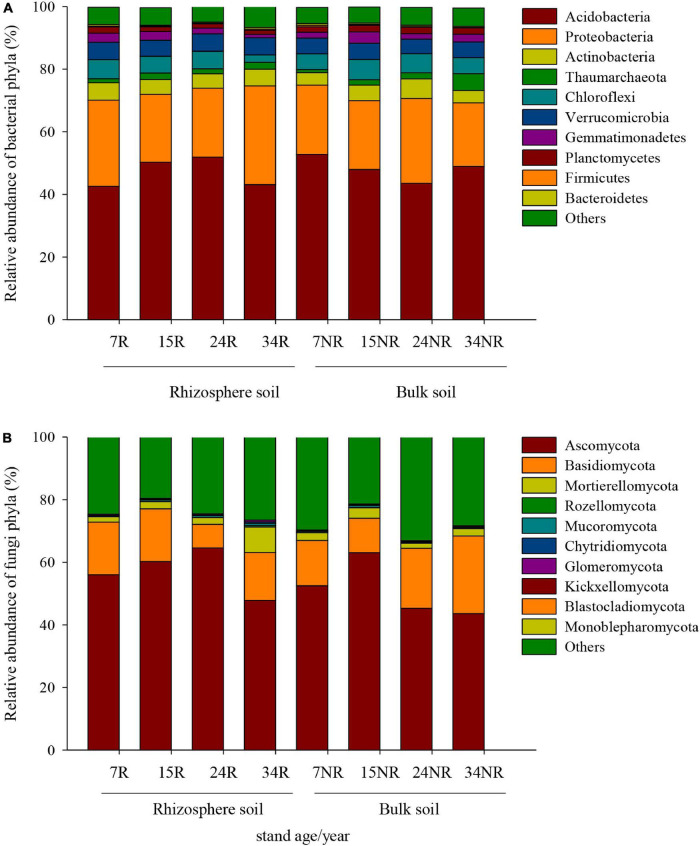
Relative abundance (%) of the most dominant bacterial **(A)** and fungal **(B)** groups at the phyla level among different stand ages in Xinkou Forest Farm, Southeastern China. Others represent unclassified groups.

**FIGURE 3:**
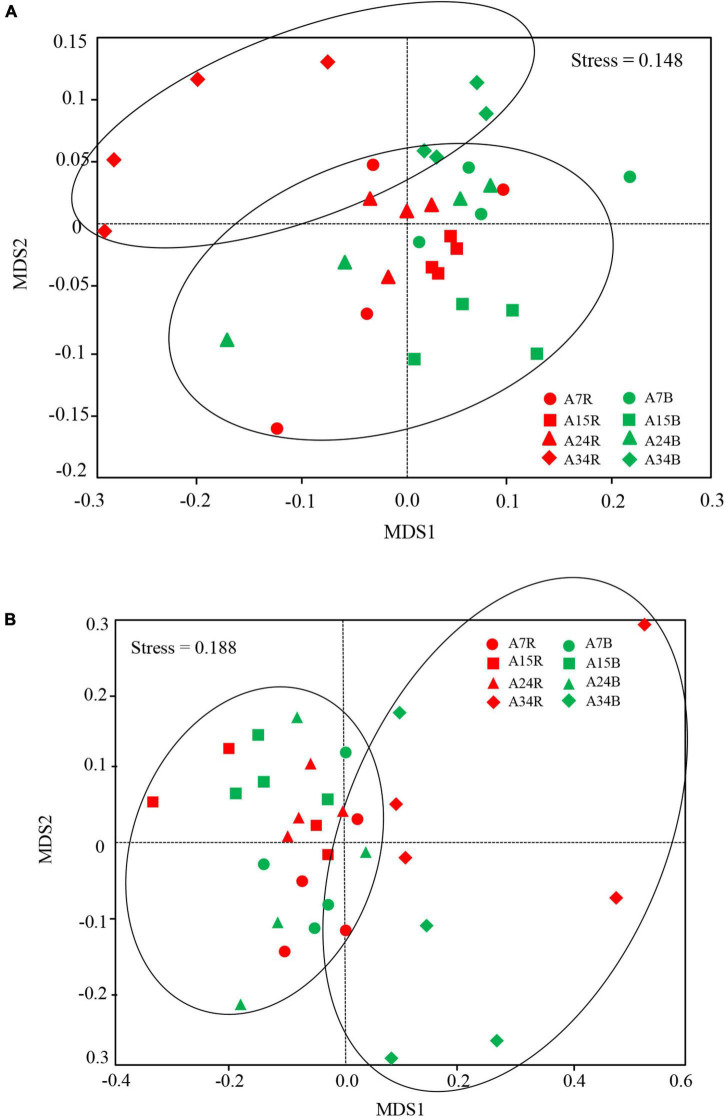
Non-metric multidimensional (NMDS) scaling plot of bacterial **(A)** and fungal **(B)** community structures across all soil samples among different stand ages in Xinkou Forest Farm, Southeastern China. Stress value is indicated in the figure. Data points are from different stand ages (A7, A15, A24, and A34) and soil locations (R, rhizosphere soil; B, bulk soil). The samples with same stand age are represented in the same shape, while the samples in rhizosphere and bulk soil are represented by red and green color.

**FIGURE 4:**
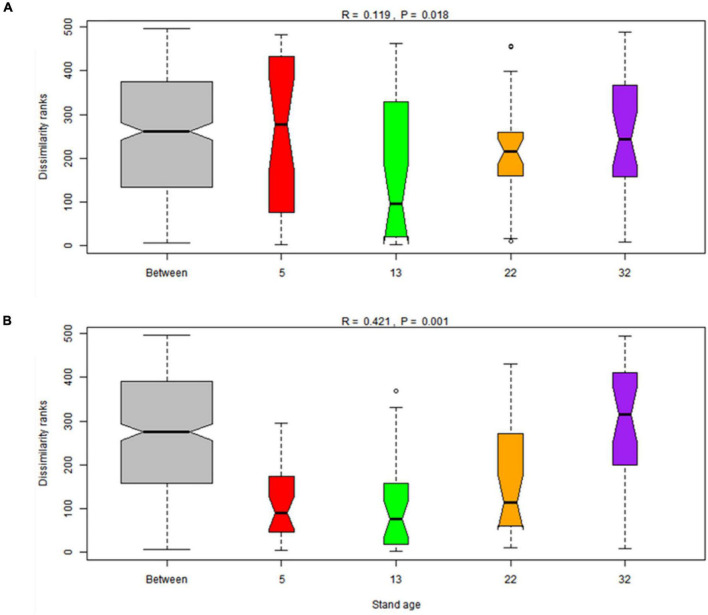
Boxplot of analysis of similarities (ANOSIM) based on the Bray–Cutis dissimilarities of soil bacterial **(A)** and fungal **(B)** community among different stand ages (7-, 15-, 24- and 34-year-old stands) in Xinkou Forest Farm, Southeastern China.

The dominant bacterial phyla at age 7, 15, 24, and 34 were *Acidobacteria* (47.7, 49.1, 47.7, and 46.0%, respectively) and *Proteobacteria* (24.8, 21.8, 24.6, and 25.9%, respectively) in rhizosphere and bulk soil, followed by *Actinobacteria* (4.8, 4.8, 5.4, and 4.6%, respectively) (Figure 2A). The relative abundance of *Acidobacteria* and *Ascomycota* followed similar trends in rhizosphere soil, which increased gradually along the age series from 7a to 24a, and then decreased at age 34 (Figure 2). However, *Acidobacteria* in bulk soil and *Proteobacteria* in rhizosphere soil showed a reversed trend, which decreased slightly from stand ages 7a to 24a, and then increased at age 34 (Figure 2A). The most abundant fungal phyla at age 7, 15, 24, and 34 were *Ascomycota* (54.2, 61.7, 54.9, and 45.7%, respectively) and *Basidiomycota* (15.7, 13.9, 13.3, and 20.1%, respectively) (Figure 2B). Other phyla such as *Rozellomycota*, *Mucoromycota*, and *Chytridiomycota* accounted only a minor proportion (<2%) of the fungal–community composition (Figure 2B). The ordination of NMDS clearly identified variations in microbial beta diversity among all the sampling sites, with no obvious separation effects of soil locations, but both bacterial and fungal groups varied significantly with stand age (Figure 3). The profiles of bacterial (Figure 3A) and fungal (Figure 3B) beta diversity at stand ages 7, 15, and 24 tended to group together and were clearly separated from those at stand age 34 site. Simultaneously, the ANOSIM was applied to reveal differences in the soil bacterial (Figure 4A) and fungal (Figure 4B) beta diversity, which indicated that there was a significant effect of plantation age on the structure of soil bacterial (*R* = 0.119, *p* < 0.05) and fungal community (*R* = 0.421, *p* < 0.01), and soil bacterial and fungal community structure differed significantly among the four stand ages (Figure 4).

### Relationships Between Soil Characteristics and Microbial Community Composition

**FIGURE 5:**
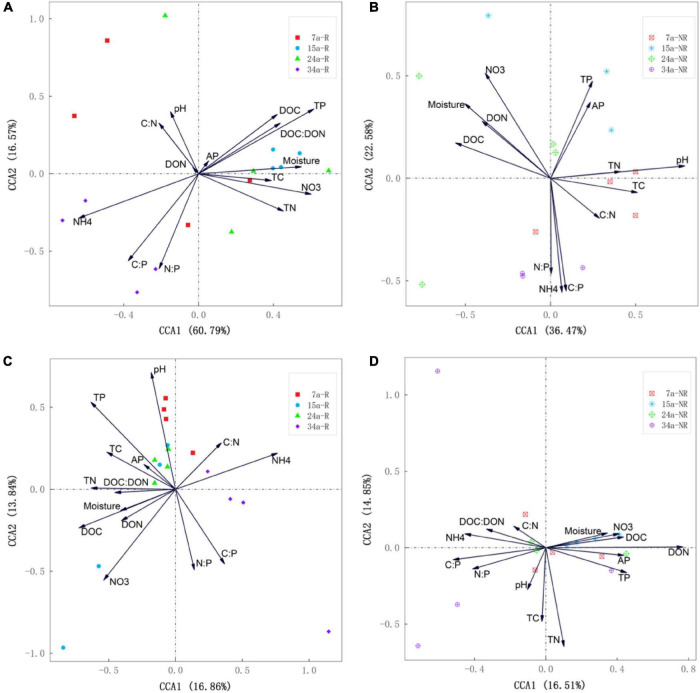
Canonical correspondence analysis (CCA) illustrating the effects of environmental factors (arrows) on rhizosphere soil bacterial **(A)** and fungal **(C)** community structure (symbols) among different stand ages. CCA illustrating the effects of environmental factors (arrows) on bulk soil bacterial **(B)** and fungal **(D)** community structure (symbols) among different stand ages. The values of axes 1 and 2 are the percentage that can be explained by the corresponding axis.

**FIGURE 6:**
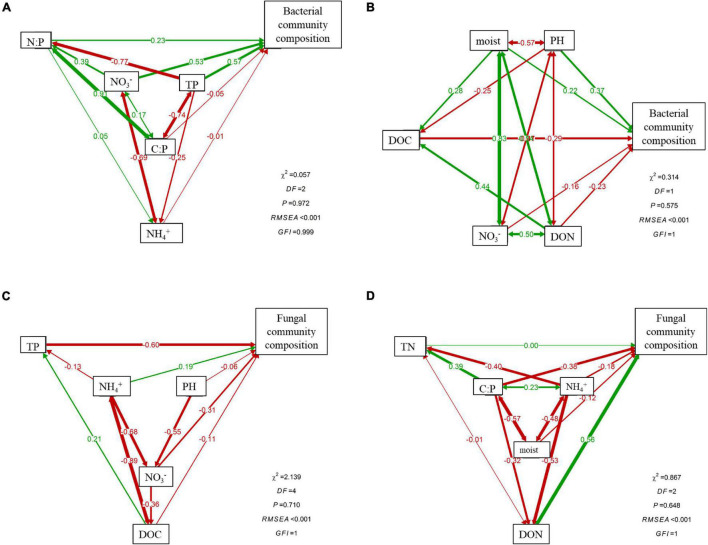
Structural equation models showing the contributions of soil properties on rhizosphere and bulk soil bacterial **(A,B)** and fungal **(C,D)** community composition. Numbers on arrows are standardized path coefficients. Arrow thickness represents the magnitude of the path coefficient. Red solid lines indicate that the standardized regression weights are negative; green solid lines indicate that the standardized regression weights are positive.

Canonical correspondence analysis (CCA) showed that environmental and nutrient variables of soil explained changes in bacterial and fungal communities well, but contrasting driving factors were identified for the dissimilarities of rhizosphere and bulk soils (Figure 5). The first two axes together explained 77.36 and 30.7% of the total variations in bacterial and fungal communities in rhizosphere soil (Figures 5A,C), while in bulk soil, the first two axes accounted for 59.05 and 31.36% of the overall variations (Figures 5B,D). During different stand ages, soil nutrient availability (TP, NH_4_^+^–N) and stoichiometric ratios (C:P) were the main contributors to the dissimilarities of bacterial community in rhizosphere, while soil pH and NO_3_^–^–N significantly drove the variations in bulk soils. Distinct controlling factors were also found for soil fungal communities in rhizosphere and bulk soil, respectively (Figures 5C,D). The SEM analysis further showed direct and indirect relationships between soil physiochemical properties and bacterial (Figures 6A,B) and fungal (Figures 6C,D) community composition. For example, soil NO_3_^–^–N and TP primarily and directly affected bacterial community composition in rhizosphere soil, while NH_4_^+^–N indirectly affected bacterial community composition by influencing NO_3_^–^–N. In bulk soil, moisture, both directly and indirectly (through pH and DOC), affected bacterial community composition. On the other hand, soil TP primarily impacted fungal community composition in rhizosphere, while DON and C:P primarily affected fungal community composition in bulk soil.

Analysis through Pearson’s correlation demonstrated that rhizosphere Shannon–Weiner diversity index of bacteria was significantly positively correlated with soil NH_4_^+^–N, but negatively correlated with soil moisture, TN, and NO_3_^–^–N (Table 3, *p* < 0.05). In bulk soil, the bacterial Shannon–Weiner index was significantly positively correlated with soil DOC but negatively correlated with soil pH (Table 3, *p* < 0.05). Soil DOC and NH_4_^+^–N had a significant impact on controlling rhizosphere fungal community diversity, while soil pH and DOC played significant roles in regulating bacterial community diversity of bulk soil (Table 3).

## Discussion

### Changes in Soil Physiochemical Properties Among Different Plantation Ages

Soil nutrient concentrations (TP, NH_4_^+^–N, NO_3_^–^–N, and DOC) were more strongly affected by stand age rather than soil location (Tables 1, 2), and these results partially agreed with previous studies (Chen et al., 2020; Wang et al., 2020), which were mainly explained by the different vegetation characteristics during stand development. The lowest soil NH_4_^+^–N and NO_3_^–^–N content were observed in 15- and 7-year-old stands, respectively, mainly because Chinese fir plantation had undergone slash burning after clear-cut harvest, leading to a small amount of undergrowth vegetation and nutrient return from litter in younger stands. Moreover, fast-growing stands had large demand for available nutrients (NH_4_^+^–N and NO_3_^–^–N), and the high intensity of rainfall also contributed to considerable nutrient erosion. In contrast, the 34a stand had the lowest content of DOC, DON, and TP, which may be due to the decrease in soil enzyme activity in older stands. It has been reported that fast-growing trees from 3 to 25 years increased soil C- and N-degrading enzyme activity [e.g., β-glucosidase (BG), cellobiohydrolase (CB), chitinase, etc.] in order to enhance mineralization and overcome nutrient stress (Lungmuana et al., 2017), but subsequent reduction in enzyme production was observed in mature stands since nutrient demands are satisfied. Soil TP significantly decreased in 34a stands compared with younger stands (7a, 15a, and 24a), while soil AP exhibited a downtrend from young to old stands (Table 2), and this phenomenon may be caused by higher P consumption in older trees (Vitousek et al., 2010). A previous study has reported that P uptake increased by 2.2–2.8 kg P ha^–1^ year^–1^ as the stands develop from age 8 to 24 (Ma et al., 2007), leading to a gradual P depletion in soil.

### Differences in Microbial Diversity Along Plantation Age Gradients Between Rhizosphere and Bulk Soil

Our results highlighted the greater role of age phases than soil locations (rhizosphere vs. bulk) in determining soil microbial community diversities (both alpha and beta diversity) in Chinese fir plantations (Figures 1, 3). This result agreed with our first hypothesis, emphasizing the importance of changes on environmental variables in shaping soil microbial communities. In our study, edaphic factors changed more markedly along stand age gradients than between rhizosphere and bulk soil (Tables 1, 2). Besides, our results indicated that the alpha diversity of bacterial community was more responsive to different stand ages than fungal community (Figure 1); similar findings were also reported by Delgado-Baquerizo et al. (2017), who observed that soil bacterial community was more sensitive than the fungal community to changes in soil nutrients. Fungal alpha diversity did not differ significantly among stand ages, possibly due to their stronger adaption to the environment compared with bacterial community (Corneo et al., 2013). For example, many fungal species can form spores, which are able to efficiently exploit nutrients particularly in oligotrophic environment (Harrison, 2005; Davison et al., 2015). Previous studies reported that arbuscular mycorrhizal fungal spores demonstrated high survival rates in harsh environmental conditions, and they might form a propagule bank that can efficiently exploit favorable conditions (Smith and Read, 2008; Varga et al., 2015). Besides, substantial propagule transport might be mediated by various dispersal agents such as wind, birds, and human activities (Egan et al., 2014). Similar changes in soil bacterial diversity indices along age gradients were also reported by other studies (Wang et al., 2019). The decrease in the alpha diversity of soil bacterial community during stand development (7a to 15a or 24a) was paralleled by the decrease in soil nutrients, particularly soil NH_4_^+^–N (Table 2). Furthermore, the fast-growing stage of *Cunninghamia* and aboveground vegetation acquire a great amount of nutrients, aggravating the competition with microbes. As stand age increases from 24a to 34a, plantations gradually reach maturity, accompanied by the accumulation of forest floor and alleviated competition. As a result, the improvement in nutrient environment may promote the diversity of bacterial community.

Surprisingly, the consistent diversity of the microbial communities for most stand ages was observed between the rhizosphere and bulk soil (Figure 1), which is contrary to the previous findings in agricultural and grassland ecosystems that microbial diversities were generally lower in the rhizosphere than bulk soil (Ai et al., 2012; Guo et al., 2016). The discrepancy may be explained by the highly acidic (4.15–4.53) environment for both rhizosphere and bulk soils in the Chinese fir plantation, unlike the aforementioned agricultural (pH 8.18–8.38) and grassland (pH 5.88–6.05) ecosystems. Mounting evidences suggest that soil pH is a key regulator for shaping the structures of microbial communities (Wang et al., 2015). Our low pH could create a strong filter for microorganisms and lead to the consistency of microbial diversities between the rhizosphere and bulk soils.

### Shifts in the Microbial Community Composition Along Age Gradients in the Rhizosphere and Bulk Soil

The soil bacterial community was dominated by *Acidobacteria*, *Proteobacteria*, and *Actinobacteria* (Figure 2A), while soil fungal community was dominated by *Ascomycota* and *Basidiomycota* across all age gradients (Figure 2B); these results were also confirmed by other studies (Nie et al., 2018; Wang et al., 2018; Liu et al., 2020). The dominant microbial phyla in the rhizosphere and bulk soil exhibited inconsistent distribution patterns as stand ages, suggesting distinct ecological strategy (r-strategist vs. k-strategist) of different microbial taxa, as well as different microenvironments (i.e., nutrient stoichiometry and root exudates) between the rhizosphere and bulk soil. For example, *Acidobacteria* are generally oligotrophic and versatile heterotrophs (Nemergut et al., 2010). The higher abundance of *Acidobacteria* was observed in the oligotrophic bulk soils (relatively lower soil DOC and nitrate content at 7a and 34a compared with 15a and 24a) further demonstrating their oligotrophic lifestyle (Figure 1A and Table 2; Fierer et al., 2007). At the early stage of plantation development, an insufficient supply of nutrients may meet the ecological strategy of *Acidobacteria* (k-strategist). As plantation developed, increased above- and below-ground biomass and decomposition of litter may improve the nutrient condition. As a result, higher abundance *Proteobacteria* (considered as copiotroph) was observed in nutrient-rich bulk soil (27.1% at 24a), showing an opposite trend to that of phylum *Acidobacteria* (Figure 1A). It is noteworthy that the relative abundance of *Acidobacteria* was approximately twofold as that of *Proteobacteria* in all samples (Figure 1A), indicating the overall nutrient-poor condition in this site. Nevertheless, no clear pattern along stand age gradients of phylum *Actinobacteria* was obtained in our study (Figure 1A), possibly due to the fact that *Actinobacteria* contained both oligotrophic and copiotrophic members (Morrissey et al., 2016).

Furthermore, *Acidobacteria* appears capable of reducing nitrate and nitrite, and possibly nitric oxide in soil (Ward et al., 2009). The different distribution patterns along stand age may indicate an altered N cycle in these soils, corresponding to the changes in the content of soil NH_4_^+^–N and NO_3_^–^–N (Figure 1 and Table 2). In bulk soil, higher abundance of *Acidobacteria* was observed in 7a and 34a stands relative to 15a and 24a stands, accompanied by a decrease in soil NO_3_^–^–N contents (Figure 1 and Table 2). Interestingly, the relative abundance of *Acidobacteria* showed a first increasing and then declining tendency from ages 7 to 34 years in the rhizosphere soil, but the opposite was true for bulk soil (Figure 2A). The reason for this trend remains unclear and requires more investigation.

The dominant phyla in fungal communities were *Ascomycota* and *Basidiomycota*, and their changes drive the difference in community structure among different age groups in the rhizosphere and bulk soil (Figure 1B). *Ascomycota* are generally accepted as cellulose decomposers or sugar fungi, which have limited ability in lignin degradation (Osono, 2007). In contrast, *Basidiomycota* are inclined to degrade recalcitrant lignin-containing material, thus, prevailing in the later litter decomposition process (Osono, 2007; Lundell et al., 2010). The decrease in *Ascomycota* and increase in *Basidiomycota* at stand ages 24a and 34a implied a shift in the soil C composition with increasing stand age. That is to say, it is likely that more recalcitrant C was gradually accumulated, and labile C was depleted in soil, resulting in an increase in lignin decomposer (*Basidiomycota*) and decrease in sugar fungi (*Ascomycota*) in older stands. The uniqueness of the morphological (tough, needle shaped) and chemical (high lignin content, slow decaying) characteristics for Chinese fir litter is likely to be the biological reason for the accumulation of more recalcitrant C in soil as stands age (Zhou et al., 2015).

The variance in community composition (Figure 2B) demonstrated shifts in fungal community as stands aged. Among the soil funguilds, mycorrhizal fungi are mostly affiliated with *Basidiomycota* and contribute to the nutrient uptake of the plant; thus, they are particularly abundant in low-nutrient environment (Read and Perez-Moreno, 2003). In rhizosphere, *Basidiomycota* was enriched in fasting-growing plantations (7R, 16.83%; 15R, 16.84%), indicating their role in facilitating the early establishment of stand and subsequent tree growth. In contrast, the free-living saprotrophic fungi mostly belong to *Ascomycota*, assisting the litter and organic matter degradation (Baldrian et al., 2011). A correlation study suggested that the relative abundance of *Ascomycota* was closely linked with soil NH_4_^+^–N and AP contents (Supplementary Table 6), highlighting their role in nutrient uptake and cycling.

### Contrasting Drivers of Bacterial and Fungal Communities Along the Age Gradients in the Rhizosphere and Bulk Soil

As expected in our second hypothesis, soil microbial communities in rhizosphere and bulk soils were driven by different edaphic factors, respectively. Specifically, rhizosphere bacterial communities were tightly associated with soil nutrient contents (e.g., TP, NH_4_^+^–N, and NO_3_^–^–N) and stoichiometric ratios (e.g., C:P and N:P) (Figures 5A, 6A), while bulk bacterial communities were only significantly correlated with soil pH and NO_3_^–^–N (Figures 5B, 6B). Similarly, the driving factors for variations in fungal communities were also location specific (rhizosphere vs. bulk soil) (Figures 5C,D, 6C,D). These findings indicate that changes in rhizosphere microbial communities were more sensitive to variations in soil nutrients than bulk soil. This result was also confirmed by the study of Delgado-Baquerizo et al. (2017).

Soil characteristics are prominent factors regulating the rhizosphere microbial community because they could influence root exudation patterns, which, in turn, affect the rhizosphere microbial community composition (Philippot et al., 2013; Ren et al., 2018). Root exudates contain low-molecular-mass compounds and sugar polymers, which could be used as carbon sources by rhizosphere microbiota (Walker et al., 2003; Philippot et al., 2013). This may also explain the remarkable higher DOC content in rhizosphere than bulk soil (Table 2).

The threshold of N-limitation for part of the microbial population is 6–8 mg of NH_4_^+^ kg^–1^ soil (Jackson et al., 1989), which also corresponded to the current study (Table 2). In this case, soil NO_3_^–^ assimilation could afford an alternative N source for the synthesis of cellular amino acids and protein (Geisseler et al., 2010). Therefore, NO_3_^–^–N also plays a role in shaping microbial community in our study (Figures 5B, 6A,C). A previous study showed that pH was a key soil factor affecting the soil microbial community (Rousk et al., 2010); our results also confirmed this (Figure 5, Table 3, and Supplementary Table 6).

## Conclusion

This study provides insight into the distributional patterns and drivers of microbial communities in rhizosphere and bulk soil along an age gradient (7–34a) and improves our understanding of microbial ecology in subtropical Chinese fir plantations. Soil bacterial, but not fungal, community diversity significantly differed among stand ages and between rhizosphere and bulk soils. Soil bacterial diversity was higher in mature than in middle-aged (fast-growing) plantation. Soil microbial community composition varied significantly with stand age but not between the rhizosphere and bulk soil, suggesting differences and diverse metabolic strategies of microorganisms to environmental changes along with stand development. We also identified specific drivers between the bacterial and fungal communities in rhizosphere and bulk soil. Nutrient availability and their stoichiometric ratios (e.g., TP, NO_3_^–^–N, C:P, and N:P) largely explained the microbial–community variations in rhizosphere soil, while pH, NO_3_^–^–N, DON, and TN accounted for most of the microbial–community variations in bulk soil.

## Data Availability Statement

The sequencing data have been deposited in the National Center for Biotechnology Information (NCBI) Sequence Read Archive (SRA), https://www.ncbi.nlm.nih.gov/bioproject/PRJNA694295.

## Author Contributions

YW, XL, XT, and YH planned and supervised the project, conceived and designed the experiments, and reviewed drafts of the manuscript. WG, DD, and YW performed the experiments, analyzed the data, and led the manuscript production. YW, PJ, and XL analyzed the data and made the charts. All authors have read and agreed to the published version of the manuscript.

## Conflict of Interest

The authors declare that the research was conducted in the absence of any commercial or financial relationships that could be construed as a potential conflict of interest.

## Publisher’s Note

All claims expressed in this article are solely those of the authors and do not necessarily represent those of their affiliated organizations, or those of the publisher, the editors and the reviewers. Any product that may be evaluated in this article, or claim that may be made by its manufacturer, is not guaranteed or endorsed by the publisher.

## Footnotes

1
http://drive5.com/uparse/

2
http://www.arb-silva.de/
