# Supplementary material for: Changes in Bulk and Rhizosphere Soil Microbial Diversity and Composition Along an Age Gradient of Chinese Fir (Cunninghamia lanceolate) Plantations in Subtropical China
Source: Front Microbiol. 2022 Feb 23;12:777862. doi: 10.3389/fmicb.2021.777862 (PMC8904968; doi:10.3389/fmicb.2021.777862)
Supplement: Supplementary file 1 [file Data_Sheet_1.docx]

Table S1Results of two-way ANOVAsshowing the effects of stand age, location (rhizosphere and bulk) and their interaction on the soil parameters.

| Factors | TC | TN | TP | Moisture | pH | DOC | DON | DOCN | NH_4_^+^-N | NO_3_^-^-N | AP |
| --- | --- | --- | --- | --- | --- | --- | --- | --- | --- | --- | --- |
| Stand age | 0.28 | 0.74 | *** | *** | *** | *** | 0.27 | *** | *** | *** | 0.06 |
| Location | 0.19 | 0.40 | 0.33 | 0.98 | *** | *** | ** | *** | ** | 0.75 | 0.32 |
| Stand age*location | 0.75 | 0.82 | 0.65 | 0.64 | 0.46 | *** | 0.22 | *** | 0.15 | 0.96 | 0.63 |

Note: Numbers in the table represented Sig.; TC, total carbon content; TN, total nitrogen content; TP, total phosphorus content; DOC, dissolved organic carbon content; DON, dissolved organic nitrogen content; DOCN, DOC/DON ratio; MBC, microbial biomass carbon; MBN, microbial biomass nitrogen; MBCN, MBC/MBN ratio; AP, available P content; ****P*< 0.001; ** *P*< 0.01; * *P*< 0.05.

Table S2 Results of one-way ANOVAs showing the effects of soil location (rhizosphere and bulk) on soil physiochemical properties and microbial alpha diversities at each sampling point.

| Soil location | Stand age | TC | TN | TP | Moisture | pH | DOC | DON | DOCN | NH_4_^+^-N | NO_3_^-^-N | AP |
| --- | --- | --- | --- | --- | --- | --- | --- | --- | --- | --- | --- | --- |
| Rhizosphere | 7a | 20.15a | 1.32a | 0.31a | 29.75a | 4.44a | 201.58a | 32.26a | 7.54a | 4.74a | 1.46a | 2.69a |
| Non-rhizosphere |  | 19.85a | 1.32a | 0.30a | 30.10a | 4.51a | 70.18b | 27.15a | 2.15b | 6.12a | 1.73a | 2.28a |
| Rhizosphere | 15a | 19.45a | 1.45a | 0.29a | 33.98a | 4.29a | 267.92a | 24.63b | 11.52a | 3.00a | 9.65a | 2.06a |
| Non-rhizosphere |  | 18.10a | 1.36a | 0.30a | 34.87a | 4.46a | 85.34b | 42.03a | 2.03b | 3.29a | 9.60a | 2.57a |
| Rhizosphere | 24a | 21.62a | 1.40a | 0.31a | 36.01a | 4.19a | 112.76a | 30.79a | 3.67a | 4.71b | 6.70a | 1.98a |
| Non-rhizosphere |  | 19.23a | 1.28a | 0.32a | 34.55a | 4.28a | 93.75a | 34.93a | 2.79a | 5.70a | 7.22a | 1.84a |
| Rhizosphere | 34a | 18.62a | 1.32a | 0.23a | 30.12a | 4.20b | 70.78a | 24.94a | 3.02a | 5.48a | 4.71a | 1.75a |
| Non-rhizosphere |  | 18.38a | 1.34a | 0.22a | 30.39a | 4.41a | 54.12a | 28.54a | 2.00a | 5.65a | 4.60a | 1.79a |

to be continued

| Bacteria | | | Fungi | | |
| --- | --- | --- | --- | --- | --- |
| Number of OTUs | Simpson’s diversity index | Shannon-Wiener index | Number of OTUs | Simpson’s diversity index | Shannon-Wiener index |
| 2680a | 0.10b | 9.18a | 1287a | 0.94a | 5.85a |
| 2174b | 8.67a | 8.66b | 1307a | 0.95a | 5.97a |
| 2215a | 8.71a | 8.71a | 1355a | 0.91a | 5.68a |
| 2246a | 8.73a | 8.72a | 1524a | 0.94a | 6.11a |
| 2308a | 8.82a | 8.82a | 1444a | 0.96a | 6.15a |
| 2386a | 8.98a | 8.98a | 1214a | 0.89a | 5.50a |
| 2470a | 9.19a | 9.18a | 1374a | 5.06a | 6.45a |
| 2039a | 8.74a | 8.74b | 1126b | 0.95b | 0.89b |

Table S3Total raw and final sequence numbers in the rhizosphere and bulk soils for each stand age of Chinese fir plantation at Xinkou Forest Farm, Fujian, China.

| Location | Stand age (years)  (years) | Sample | Total Raw 16S *r*RNA sequences | Clean 16S *r*RNA sequences | OTUs  3%cut-off | Total Raw ITS sequences | CleanITS sequences | OTUs  3%cut-off |
| --- | --- | --- | --- | --- | --- | --- | --- | --- |
| Rhizosphere | 7 | A7R1 | 97678 | 64415 | 3066 | 69561 | 65918 | 1173 |
|  |  | A7R2 | 87338 | 56454 | 2953 | 67041 | 66395 | 1268 |
|  |  | A7R3 | 80333 | 53158 | 2303 | 65115 | 61604 | 1431 |
|  |  | A7R4 | 94731 | 64272 | 2399 | 73175 | 69854 | 1274 |
|  | 15 | A15R1 | 83143 | 62408 | 2407 | 65248 | 63359 | 1498 |
|  |  | A15R2 | 96679 | 65096 | 2303 | 75884 | 69835 | 1374 |
|  |  | A15R3 | 81909 | 55564 | 2116 | 66004 | 61885 | 992 |
|  |  | A15R4 | 86112 | 51641 | 2032 | 68179 | 66588 | 1555 |
|  | 24 | A24R1 | 95498 | 60642 | 2228 | 69198 | 65891 | 1492 |
|  |  | A24R2 | 77768 | 50581 | 2087 | 62932 | 61738 | 1233 |
|  |  | A24R3 | 92494 | 59412 | 2070 | 62434 | 60339 | 1299 |
|  |  | A24R4 | 87629 | 55931 | 2845 | 54220 | 52206 | 1750 |
|  | 34 | A34R1 | 81725 | 59522 | 2864 | 69223 | 63647 | 1194 |
|  |  | A34R2 | 74810 | 52740 | 2674 | 71500 | 63935 | 1342 |
|  |  | A34R3 | 72068 | 46596 | 2171 | 62731 | 61168 | 1525 |
|  |  | A34R4 | 68152 | 48132 | 2172 | 62611 | 60774 | 1433 |
| Bulk | 7 | A7NR1 | 86624 | 62337 | 2358 | 62866 | 60482 | 1182 |
|  |  | A7NR2 | 84439 | 61434 | 2192 | 66749 | 63562 | 1069 |
|  |  | A7NR3 | 81395 | 69063 | 2067 | 68470 | 65030 | 1822 |
|  |  | A7NR4 | 76886 | 48791 | 2079 | 69103 | 65189 | 1154 |
|  | 15 | A15NR1 | 55213 | 41707 | 1976 | 61136 | 58265 | 1733 |
|  |  | A15NR2 | 55478 | 34281 | 2209 | 68690 | 64893 | 1962 |
|  |  | A15NR3 | 77560 | 49966 | 2056 | 71332 | 68821 | 1206 |
|  |  | A15NR4 | 84212 | 55814 | 2744 | 65053 | 60316 | 1193 |
|  | 24 | A24NR1 | 85560 | 59064 | 2882 | 69502 | 65703 | 1248 |
|  |  | A24NR2 | 77399 | 54089 | 2552 | 66113 | 62191 | 1233 |
|  |  | A24NR3 | 60630 | 39362 | 2034 | 69979 | 67700 | 970 |
|  |  | A24NR4 | 64729 | 42049 | 2077 | 63629 | 61328 | 1406 |
|  | 34 | A34NR1 | 68030 | 50165 | 2202 | 65493 | 61893 | 1100 |
|  |  | A34NR2 | 65249 | 45354 | 2028 | 67280 | 63568 | 1141 |
|  |  | A34NR3 | 60520 | 40272 | 1905 | 61556 | 58124 | 1288 |
|  |  | A34NR4 | 75978 | 44559 | 2019 | 66024 | 63010 | 973 |

Note:sample name for each stand age (A7, A15, A24, A34), R, rhizosphere; NR, non-rhizosphere; and the number after site location means the plot number.

Table S4 Results of two-way ANOVAs showing the effects of stand age, location (rhizosphere and bulk) and their interaction on microbial alpha diversity.

| Factors | Bacteria | | | Fungi | | |
| --- | --- | --- | --- | --- | --- | --- |
|  | Number of OTUs | Simpson’s diversity index | Shannon-Wiener index | Number of OTUs | Simpson’s diversity index | Shannon-Wiener index |
| Stand age  Location  Stand age*location | 0.57  0.07  0.15 | ***  **  * | 0.19  *  * | 0.46  0.41  0.27 | 0.81  0.14  0.05 | 0.96  0.13  0.05 |

Note: Numbers in the table represented Sig.; ****P*< 0.001; ** *P*< 0.01; * *P*< 0.05.

Table S5 Pearson correlation coefficients between bacterial, fungal alpha diversity and environmental variables.

|  | TC | TN | TP | Moisture | pH | DOC | DON | DOCN | NH_4_^+^-N | NO_3_^-^-N | AP |
| --- | --- | --- | --- | --- | --- | --- | --- | --- | --- | --- | --- |
| **Bacteria**  Number of OTUs  Simpson’s diversity index  Shannon-Wiener index  **Fungi**  Number of OTUs  Simpson’s diversity index  Shannon-Wiener index | -0.33  -0.14  -0.14  -0.08  -0.05  -0.12 | -0.49  -0.23  -0.23  -0.03  -0.16  -0.19 | -0.07  **-0.49****  -0.29  0.34  0.13  0.21 | -0.05  -0.28  -0.16  -0.01  -0.01  -0.07 | -0.16  **-0.38***  -0.26  0.16  0.08  0.06 | 0.23  0.01  0.01  -0.07  -0.14  -0.13 | 0.03  -0.11  -0.11  -0.07  -0.24  -0.24 | 0.20  -0.01  -0.01  0.01  0.03  0.03 | -0.001  0.33  0.33  -0.17  0.09  0.05 | -0.23  -0.34  -0.34  0.10  -0.21  -0.11 | 0.02  -0.20  -0.11  -0.03  0.11  -0.01 |

Note: TC, total carbon content; TN, total nitrogen content; TP, total phosphorus content; DOC, dissolved organic carbon content; DON, dissolved organic nitrogen content; DOCN, DOC/DON ratio; AP, available P content; ****P*< 0.001; ** *P*< 0.01; * *P*< 0.05.

Table S6 Pearson correlation coefficients between bacterial, fungal dominant phyla and environmental variables.

|  | TC | TN | TP | Moisture | pH | DOC | DON | DOCN | NH_4_^+^-N | NO_3_^-^-N | AP |
| --- | --- | --- | --- | --- | --- | --- | --- | --- | --- | --- | --- |
| **Bacteria phyla**  *Acidobacteria*  *Proteobacteria*  *Actinobacteria*  *Thaumarchaeota*  *Chloroflexi*  **Fungi phyla**  *Ascomycota*  *Basidiomycota*  *Mortierellomycota*  *Mucoromycota*  *Glomeromycota* | **0.38***  -0.31  -0.23  -0.17  0.20  0.08  -0.06  **-0.37***  -0.20  -0.25 | **0.40***  **-0.41***  -0.19  0.04  0.08  0.25  -0.13  **-0.42***  -0.22  -0.31 | 0.24  -0.22  -0.03  **-0.47****  **0.45***  0.17  -0.28  **-0.41***  -0.03  **-0.40*** | 0.13  -0.14  0.20  -0.14  0.23  0.31  -0.16  -0.24  0.17  **-0.39*** | -0.17  **-0.35***  -0.34  0.08  0.29  -0.07  0.03  -0.28  -0.19  -0.17 | -0.11  0.06  0.22  -0.23  0.18  0.26  0.03  -0.18  -0.07  -0.14 | -0.15  0.14  **0.40***  -0.34  0.16  0.01  -0.07  -0.21  -0.03  -0.22 | -0.004  -0.03  0.02  -0.13  0.10  0.24  -0.01  -0.09  0.02  -0.06 | -0.08  0.16  -0.08  0.12  -0.17  **-0.50****  0.17  0.17  -0.24  0.23 | 0.09  -0.15  0.18  0.01  0.17  0.28  -0.08  -0.10  0.22  -0.29 | 0.18  -0.06  0.01  -0.22  -0.03  **0.38***  -0.19  -0.23  -0.17  -0.26 |

Note: TC, total carbon content; TN, total nitrogen content; TP, total phosphorus content; DOC, dissolved organic carbon content; DON, dissolved organic nitrogen content; DOCN, DOC/DON ratio; AP, available P content; ****P*< 0.001; ** *P*< 0.01; * *P*< 0.05.
